# Systemic immune inflammation index and systemic inflammation response index predict frailty progression in older patients undergoing elective orthopedic surgery

**DOI:** 10.3389/fsurg.2025.1543671

**Published:** 2025-07-09

**Authors:** Guanghan Gao, Zitian Zheng, Fei Wang, Yaonan Zhang, Lei Shi, Lin Wang, Hongyu Wang, Qingyun Xue

**Affiliations:** ^1^ Department of Orthopedics, Beijing Hospital, National Center of Gerontology, Institute of Geriatric Medicine, Chinese Academy of Medical Sciences and Peking Union Medical College, Beijing, China; ^2^ Department of Orthopedics, Beijing Hospital, National Center of Gerontology; Institute of Geriatric Medicine, Chinese Academy of Medical Sciences, Beijing, China

**Keywords:** older, frailty, SII, SIRI, orthopedic

## Abstract

**Introduction:**

The frailty status of older patients undergoing elective orthopedic surgery significantly influences their surgical benefits. Evaluating the progression of postoperative frailty assists clinicians in making informed clinical decisions. The biomarkers systemic immune inflammation index (SII) and systemic inflammation response index (SIRI), which reflect chronic inflammation and immune status, may play a positive role in predicting the progression of frailty.

**Methods:**

We conducted a single-center prospective cohort study, including patients aged 65 and older who underwent elective orthopedic surgery for chronic degenerative conditions between January 2020 and January 2022. Basic patient information, laboratory results, and frailty assessments were collected. LASSO regression was used to identify important predictive variables, and multivariate logistic regression was employed to assess the associations between SII/SIRI and frailty progression. Restricted cubic splines (RCS) were used to detect potential non-linear relationships between them. ROC curves and AUC values were utilized to assess their predictive performance. Finally, we presented stratified analyses and interaction tests of covariates.

**Results:**

A total of 546 patients were included, with 109 (19.5%) experiencing postoperative frailty progression. Multivariate regression analysis revealed that ln.SII and ln.SIRI were positively correlated with frailty progression in the fully adjusted model, with odds ratios (OR) of 3.449 and 3.084, respectively. These findings were consistent across various subgroups. The linear trend between SII–SIRI pattern/SII/SIRI levels and frailty progression was statistically significant. However, the RCS curve indicated that the non-linear model significantly outperformed the linear model. The AUC values for ln.SII, ln.SIRI, and their combined model were 0.686, 0.710, and 0.723, respectively. The cutoff values for ln.SII and ln.SIRI were 5.93 and 0.10, respectively.

**Conclusion:**

SII and SIRI can effectively serve as non-invasive preoperative screening tools for identifying older patients with chronic degenerative orthopedic diseases who are at high risk of frailty progression following elective surgical procedures.

**Clinical Trial Registration:**

https://www.chictr.org.cn/, identifier chiCTR1800018840 (Date: 2018-10-13).

## 1 Introduction

Frailty is characterized by a decline in the function of multiple physiological systems, leading to a diminished capacity to adapt to environmental changes (1, 2). This decline in physical function, which includes the inability to independently perform activities of daily living, is particularly prominent among older patients with orthopedic conditions (3). Many patients often experience a reduction in mobility, a decrease in physiological reserves, and even multisystem dysfunction due to the burden of orthopedic diseases, which can subsequently lead to frailty. These pathological manifestations may also increase intraoperative risks and contribute to perioperative and postoperative complications (4). Therefore, assessing and predicting frailty status prior to elective orthopedic surgery is crucial for making surgical decisions, determining the suitability of a patient for surgery, and selecting the optimal surgical approach (5). For patients identified as being at high risk of frailty, clinicians can implement preventive measures in advance to reduce the risk of postoperative complications, such as improving nutritional status, managing chronic diseases, or increasing bone density (6).

The primary factor accelerating the progression of frailty is age. Other risk factors include sedentary behavior, smoking, obesity, low income, and low educational level (7, 8). Notably, comorbidities such as osteoporosis or hyperlipidemia have emerged as independent risk or protective factors for future frailty progression (9). In terms of indicators reflecting a patient’s frailty status, nutritional status, gait speed, and grip strength can effectively reflect the state of frailty (9–13). Furthermore, substantial evidence suggests that inflammation and immune dysregulation are associated with frailty (9, 14, 15). The systemic immune inflammation index (SII) and the systemic inflammation response index (SIRI) are composite indices calculated based on routine blood test results, incorporating neutrophil, lymphocyte, monocyte, and platelet counts. These comprehensive and innovative inflammatory biomarkers have recently been proposed, based on immune cell subsets and platelet aggregation (15–17). These indices have been widely used to study the associations between chronic inflammatory states and various human diseases, including cancer, metabolic disorders, and inflammatory diseases (18, 19). However, their predictive role in frailty remains unclear.

For the specific population of older patients undergoing elective orthopedic surgery, understanding whether postoperative frailty progresses is of greater clinical significance than merely observing frailty status at a single time point, due to the impact of surgical trauma and the subsequent rehabilitation period. Currently, research and evaluation of frailty status in patients following elective orthopedic surgery are limited, and there is a lack of effective biomarkers for predicting postoperative frailty progression. This poses challenges for optimizing surgical decision-making and perioperative management in older orthopedic patients. This study aims to investigate the relationship between SII/SIRI and the progression of postoperative frailty in older patients undergoing elective orthopedic surgery and to explore their predictive value for frailty progression.

## 2 Materials and methods

### 2.1 Population

In this single-center prospective cohort study, we enrolled consecutive patients aged ≥65 years undergoing elective orthopedic surgery (joint replacement or lumbar fusion) for chronic degenerative conditions (knee/hip osteoarthritis or lumbar degeneration) between January 2020 and January 2022. Patients were excluded if they had a history of recent bleeding, anemia, tumors, immune system disease, or acute trauma. Patients were randomized into two treatment groups according to a pre-generated random number table: One group served as the observation control group (observation group), while the other group received oral calcitriol 0.25 µg twice daily (bid) for a duration of 1 year (intervention group). Clinical data and frailty assessments were collected at patient admission and 3, 6, and 12 months after discharge. Patients who did not complete 1 year of calcitriol therapy, with cognitive decline and incomplete clinical follow-up data, were also excluded. All patients provided informed consent and agreed to participate in the follow-up.

### 2.2 Methods

This study was registered in the Chinese Clinical Trial Registry (ChiCTR; clinical trial number: ChiCTR1800018840), which is a primary register of the WHO International Clinical Trials Registry Platform (ICTRP). This study was also approved by the ethics committee of our hospital (2018BJYYEC-031-01), and informed consent was obtained from all participants.

The surgeries were performed by senior chief physicians at our center. All patients received standardized enhanced recovery after surgery (ERAS) protocols during hospitalization, which included standardized pain management, uniform wound care procedures, early mobilization, and procedure-specific functional training. At discharge, all patients were given printed rehabilitation guidelines and personalized exercise prescriptions.

#### 2.2.1 Study variables

Basic patient information was collected, including treatment group, gender, age, body mass index (BMI), type of surgery (joint replacement or lumbar spinal fusion), and past medical history. Routine preoperative laboratory tests, including red blood cell count (RBC), hemoglobin (HGB), white blood cell count (WBC), lymphocyte count (LYMPH), monocyte count (MON), neutrophil count (NEU), platelet count (PLT), serum total protein (TP), serum albumin (ALB), blood urea nitrogen (BUN), creatinine (CREA), and bone metabolism marker tests, including total Type I collagen amino acid extension peptide (PⅠNP), osteocalcin (OST), β-CrossLaps (β-Cross), and 25-hydroxyvitamin D (25-OH-VD), were performed. The SII was calculated using the following formula: platelet count × neutrophil count / lymphocyte count. The SIRI was calculated using the following formula: monocyte count × neutrophil count / lymphocyte count (20). To account for the positive skewness of this marker, the SII and SIRI values were log-transformed to ln.SII and ln.SIRI and analyzed as independent variables. We measured grip strength using a hydraulic hand dynamometer (JAMAR Hydraulic Hand Dynamometer, Model 5030J1; Performance Health Supply, Inc., United States). The participants sat in a chair with their elbows bent at right angles at their sides. Each hand was tested three times, with 15–30 s between each trial. The results were averaged, and the average of the higher grip strengths of the two hands was selected as the result of grip strength. In accordance with the Asian Working Group for Sarcopenia (AWGS) 2019, we defined decreased grip strength in patients whose grip strength was <28 kg for men and 18 kg for women and defined it as a categorical variable (21). Intraoperative blood loss was visually estimated by calculating the sum of the blood in suction canisters on surgical drapes and in gauzes. In addition, postoperative drainage, intraoperative transfusion status, and the preoperative American Society of Anesthesiologists (ASA) classification data were also collected.

We assessed frailty using the fatigue, resistance, ambulation, illness, and loss of weight (FRAIL) scale preoperatively and at the 3-, 6-, and 12-month follow-ups. The FRAIL scale is a simple tool used to assess frailty in older adults. The total score ranges from 0 to 5, with a score of 3 or higher indicating frailty (22). In this study, we focused on assessing the progression of frailty in older patients after elective orthopedic surgery. Patients whose postoperative frailty scores at 3, 6, and 12 months were higher than their preoperative scores were defined as having frailty progression. For example, if a patient’s preoperative frailty score was 2 and their postoperative frailty scores at the three follow-ups were 4, 5, and 3, respectively, this patient was defined as having frailty progression. Non-progression was defined as any postoperative frailty score that was no higher than the preoperative score.

#### 2.2.2 Statistical methods

All analyses were performed using R (version 4.4.2). A two-sided hypothesis test was used, with *p* < 0.05 indicating statistical significance. Continuous variables that followed a normal distribution were expressed as means ± standard deviations (SD). Non-normally distributed continuous variables are expressed as medians and interquartile ranges [median (Q1, Q3)]. Categorical variables were expressed as frequency and percentage. For patients who did not complete the grip strength test, interpolation was used to handle the missing values. Baseline characteristics between different groups were compared using the Kruskal–Wallis *H* test and Rao–Scott chi-square test, as appropriate. To categorize participants into different clusters based on their SII and SIRI measurements, we first scaled the SII and SIRI data and then applied the *k*-means method (23). Categorical analyses of SII and SIRI levels were performed individually by categorizing patients into four groups based on the tertiles of ln.SII and ln.SIRI levels. In addition, patients were divided into three groups (low, medium, and high) based on the SII–SIRI pattern, which was defined by the *k*-means algorithm.

Additionally, the least absolute shrinkage and selection operator (LASSO) regression was used to model and screen the collected variables for importance. The dependent variable was the occurrence of postoperative frailty progression, and the independent variables included treatment group, gender, age, BMI, RBC, HGB, WBC, LYMPH, MON, NEU, PLT, TP, ALB, BUN, CREA, PⅠNP, OST, β-Cross, 25-OH-VD, ASA classification, grip strength decrease, intraoperative blood loss, postoperative drainage, intraoperative transfusion status, type of operation, smoking, alcohol use, ln.SII, and ln.SIRI. The optimal regularization parameter lambda (*λ*) was selected through 10-fold cross-validation. The LASSO regression results indicated that ln.SII and ln.SIRI values were identified as valuable predictive variables for frailty progression. Subsequently, we utilized ln.SII and ln.SIRI to construct a logistic regression model. The diagnostic accuracy of the logistic regression model was evaluated using the receiver operating characteristic (ROC) curve and the area under the curve (AUC). The optimal cutoff value was determined by the maximum Youden index, which balances sensitivity and specificity. When the variable exceeds this cutoff value, this suggests a higher likelihood of the patient being diagnosed with frailty progression.

In the logistic regression model, the crude model was adjusted for no covariates. The minimally adjusted model was adjusted for treatment group, gender, age, and type of operation. In addition, all independent variables were adjusted in the fully adjusted model, except for the independent variable of interest itself and the variables required for its calculation. Multivariate logistic regression models were used to calculate odds ratios (OR) and confidence intervals (CIs) to assess the associations between SII–SIRI pattern/SII/SIRI levels and frailty progression.

Furthermore, restricted cubic splines (RCS) were used to detect potential non-linear relationships between ln.SII/ln.SIRI and frailty progression in the minimally adjusted model. We also performed multicollinearity analysis for the independent variables in minimally adjusted and fully adjusted models. In accordance with the “events per variable (EPV)” principle in statistics, a minimum of 10 outcome events is required for each variable included in the logistic regression analysis (24). Therefore, the sample size of this study is deemed sufficient.

Finally, stratified analyses by gender (male, female), age (≤74, >74 years old; grouped by the median age), type of operation (joint replacement, lumbar spinal fusion), grip strength decrease (with, without), and treatment group (observation, intervention) were performed in the minimally adjusted model, as well as interaction analyses between various stratification factors and ln.SII/ln.SIRI.

## 3 Results

### 3.1 Patient enrollment

**Figure 1:**
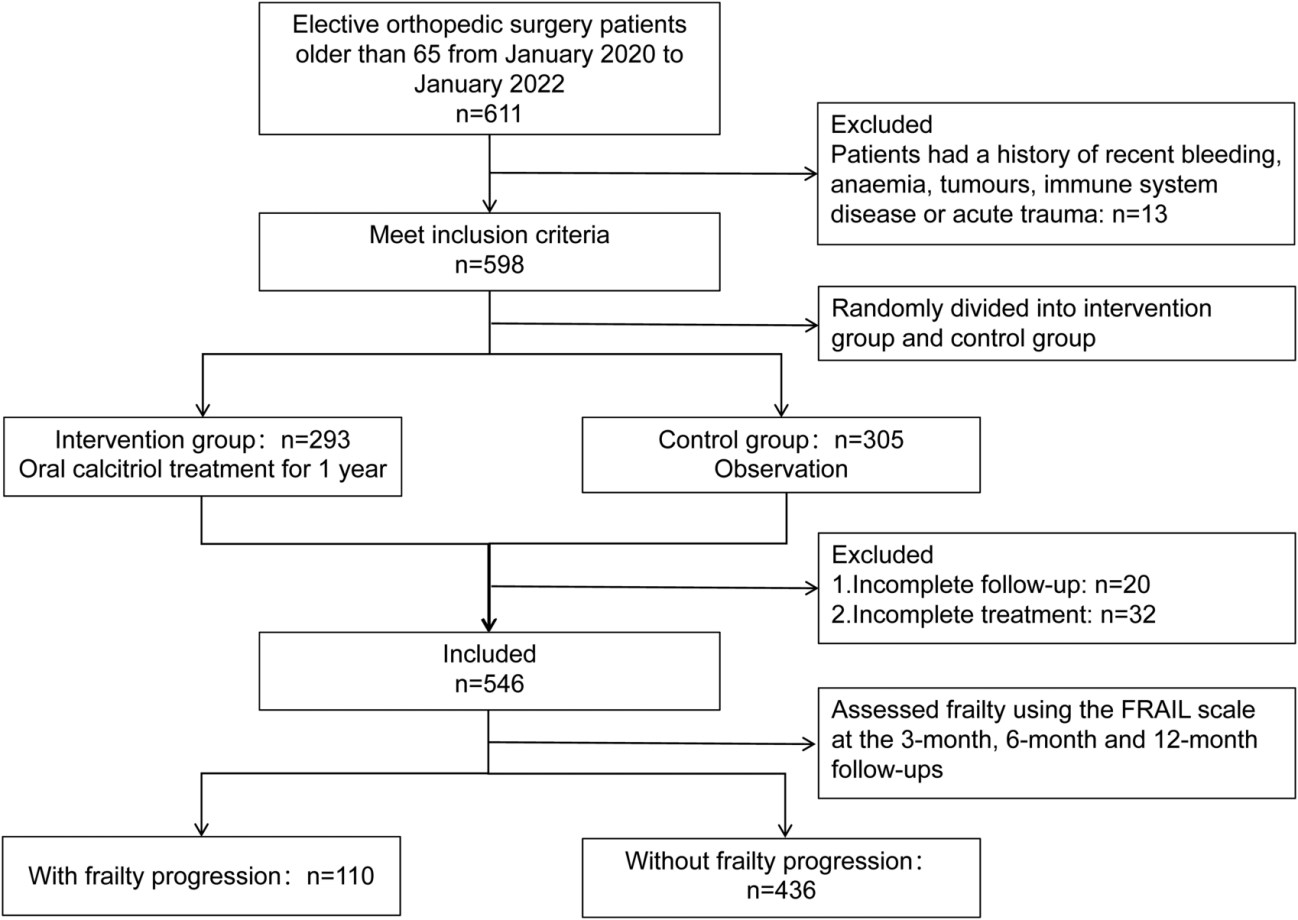
Patient enrollment.

A total of 611 patients aged 65 and above underwent elective orthopedic surgery for chronic degenerative orthopedic diseases at our center between January 2020 and January 2022. Among them, 13 patients were excluded due to anemia, tumors, or immune system diseases. A total of 598 patients were randomly assigned to either the intervention group (*n* = 293) or the observation group (*n* = 305). Over the course of the 1-year follow-up period, 20 patients were excluded due to failure to complete the follow-up, and an additional 32 patients were excluded for not completing the intervention treatment. Therefore, 546 patients were included in this study (354 females, 192 males). A total of 109 patients (20.0%) belonged to the group with postoperative frailty progression, while 437 patients (80.0%) belonged to the non-progressive group. The enrollment status of the cohort is illustrated in Figure 1. The mean follow-up time was 14 months. There were significant differences in RBC, HGB, WBC, LYMPH, NEU, PLT, TP, ALB, ln.SII, ln.SIRI, and SII–SIRI pattern (Table 1). No complications, such as periprosthetic fracture or postoperative infection, occurred in any of the patients during the 1-year follow-up period.

**Table 1:** Baseline data of older patients after elective orthopedic surgery.

Variables	General population (*n* = 546)	Non-progression (*n* = 437)	Progression (*n* = 109)	*p*
Treatment group				0.731
Observation	285 (52.2%)	226 (51.7%)	59 (54.1%)	
Intervention	261 (41.8%)	211 (48.3%)	50 (45.9%)	
Gender (*n*/%)				0.682
Male	192 (35.2%)	156 (35.7%)	36 (33.0%)	
Female	354 (64.8%)	281 (64.3%)	73 (67.0%)	
Age (year)	74 (69, 80)	74 (69, 80)	75 (70, 81)	0.150
BMI (kg/m^2^)	24.97 (23, 27.06)	25 (23.35, 27.12)	24.48 (22.06, 26.44)	0.072
Operation (*n*/%)				0.573
Joint	220 (40.3%)	173 (39.6%)	47 (43.1%)	
Lumbar	326 (59.7%)	264 (60.4)	62 (56.9%)	
RBC (*10^9^/L)	4.14 (3.79, 4.44)	4.17 (3.82, 4.45)	4.03 (3.69, 4.28)	0.013
HGB (g/L)	126.17 (117, 136)	127 (118, 137)	123 (113, 132)	0.007
WBC (*10^9^/L)	6.1 (5.11, 7.34)	6.01 (5.01, 7.12)	6.43 (5.43, 8)	0.003
LYMPH (*10^9^/L)	1.68 (1.27, 2.16)	1.76 (1.35, 2.2)	1.42 (1.13, 1.82)	<0.001
MON (*10^9^/L)	0.55 (0.45, 0.75)	0.54 (0.45, 0.72)	0.58 (0.46, 0.85)	0.102
NEU (*10^9^/L)	3.58 (2.87, 4.76)	3.5 (2.78, 4.47)	4.45 (3.17, 6.29)	<0.001
PLT (*10^9^/L)	202 (167, 240)	201 (165, 234)	211 (182, 250)	0.018
TP (g/L)	64 (61, 68)	64.24 (62, 68)	64 (61, 67)	0.042
ALB (g/L)	38 (37, 40)	38 (37, 40)	38 (36, 40)	0.042
BUN (mmol/L)	5.89 (4.95, 7.32)	5.91 (4.95, 7.3)	5.87 (4.86, 7.44)	0.794
CREA (μmol/L)	65.5 (56, 79)	66 (56, 79)	65 (56, 77)	0.306
PⅠNP (ng/ml)	48.82 (38.77, 62.49)	48.79 (39.18, 61.5)	48.97 (38.27, 64.92)	0.878
OST (μg/L)	14.69 (12.45, 18.25)	14.7 (12.5, 18.26)	14.31 (12.2, 18.24)	0.473
β-Cross (μg/L)	0.53 (0.43, 0.68)	0.52 (0.43, 0.66)	0.55 (0.45, 0.73)	0.087
25-OH-VD (ng/ml)	17.29 (14, 20.74)	17.29 (14.2, 20.8)	17.16 (13.6, 19.87)	0.538
ASA (*n*/%)				0.241
I, II	463 (84.8%)	375 (85.8%)	88 (80.7%)	
III	83 (15.2%)	62 (14.2%)	21 (19.3%)	
Decreased grip strength (*n*/%)	322 (59.0%)	256 (58.6%)	66 (60.6%)	0.791
Blood loss (ml)	200 (100, 400)	200 (100, 400)	200 (150, 400)	0.810
Drainage (ml)	300 (120, 508.54)	300 (120, 500)	240 (100, 630)	0.666
Transfusion (*n*/%)	192 (35.2%)	160 (36.6%)	32 (29.4%)	0.191
Smoking (*n*/%)	62 (11)	45 (10)	17 (16)	0.164
Alcohol (*n*/%)	79 (14)	58 (13)	21 (19)	0.150
ln.SII	6.05 (5.63, 6.52)	5.96 (5.57, 6.38)	6.5 (5.99, 6.94)	<0.001
ln.SIRI	0.21 (−0.2, 0.76)	0.09 (−0.29, 0.58)	0.76 (0.18, 1.31)	<0.001
SII–SIRI pattern (*n*/%)				<0.001
Low	202 (37.0%)	186 (42.6%)	16 (14.7%)	
Medium	241 (44.1%)	191 (43.7%)	50 (45.9%)	
High	103 (18.9%)	60 (13.7%)	43 (39.4%)	

The *p*-value is the comparative analysis result between the non-progression group and the progression group, and *p* < 0.05 indicates that the difference is statistically significant.

### 3.2 LASSO and logistic regression analysis

**Figure 2:**
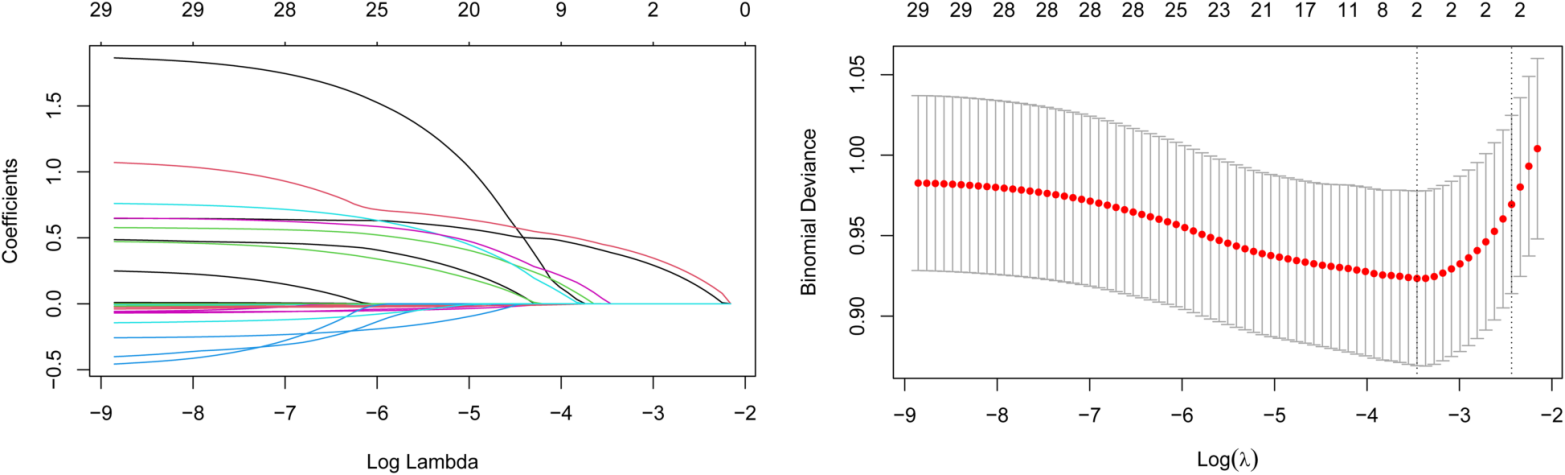
Changes in variable correlations in the LASSO regression model. **(A)** ln.SII and ln.SIR were the two variables where the coefficient curve finally drops to 0; **(B)** The optimal value of *λ* was determined through cross-validation to identify the two most critical variables.

The variation of LASSO coefficients with respect to log(*λ*) was shown in Figure 2A. The LASSO regression results demonstrated that the model was most simplified and efficient when the *λ* value corresponding to one standard error (1SE) was selected (Figure 2). Among the various variables included, the ln.SII and ln.SIRI values were identified as the most valuable predictors for frailty progression, with coefficient values of 0.101 and 0.166, respectively. We performed binary logistic regression according to the two selected variables (ln.SII and ln.SIRI). We found no multicollinearity between the independent variables (the VIF value of each variable was <5) in both minimally and fully adjusted models. In the fully adjusted model, ln.SII and ln.SIRI, as continuous variables, were consistently positively correlated with frailty progression, with OR values of 3.449 (95% CI: 2.215, 5.370; *p* < 0.001) and 3.084 (95% CI: 2.123, 4.481; *p* < 0.001), respectively.

**Figure 3:**
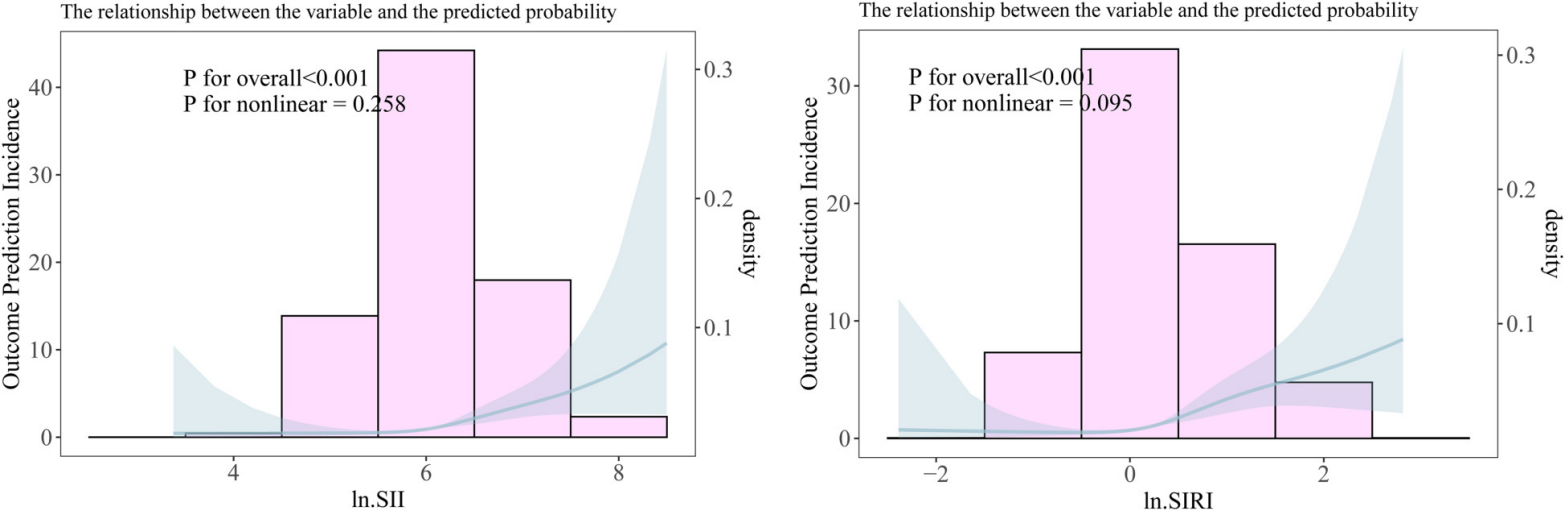
The non-linear relationship between ln.SII/ ln.SIRI and frailty progression. **(A)** The non-linear relationship between ln.SII and frailty progression. **(B)** The non-linear relationship between ln.SIRI and frailty progression.

When converting the continuous variables ln.SII and ln.SIRI into categorical variables, a similar positive association still existed in all adjustment models. The linear trend between SII–SIRI pattern/SII/SIRI levels and frailty progression was statistically significant (Table 2). Compared to the lowest-level group (Q1) of ln.SII and ln.SIRI, the OR values were 6.974 (95% CI: 3.155, 15.415) and 9.893 (95% CI: 4.330, 22.600) in the highest-level group (Q4) in the fully adjusted model, all of which were statistically significant. Similarly, significant results were observed for the SII–SIRI pattern (medium vs. low: OR = 3.693, 95% CI: 1.951, 6.989; high vs. low: OR = 11.261, 95% CI: 5.064, 25.043). However, in the RCS curve analysis (Figure 3), the likelihood ratio test indicated that the non-linear model significantly outperformed the linear model (*p* < 0.001), highlighting the importance of RCS curve analysis in our study.

**Table 2:** Linear trend test of ln.SII/ln.SIRI and postoperative frailty progression in older patients after elective orthopedic surgery.

Variables	Unadjusted		Minimally adjusted		Fully adjusted	
	OR (95% CI)	*P*	OR (95% CI)	*p*	OR (95% CI)	*p*
ln.SII	2.828 (2.044, 3.913)	<0.001	3.084 (2.154, 4.417)	<0.001	3.449 (2.215, 5.370)	<0.001
Q1 (≤5.63)	1		1		1	
Q2 (5.63, 6.05]	1.645 (0.782, 3.456)	0.189	1.660 (0.788, 3.495)	0.182	1.677 (0.774, 3.635)	0.190
Q3 (6.05, 6.52]	1.825 (0.878, 3.791)	0.107	1.859 (0.894, 3.868)	0.097	1.916 (0.887, 4.140)	0.098
Q4 (>6.52)	6.281 (3.225, 12.235)	<0.001	6.785 (3.394, 13.564)	<0.001	6.974 (3.155, 15.415)	<0.001
Linear trend	1.859 (1.507, 2.295)	<0.001	1.885 (1.512, 2.349)	<0.001	1.861 (1.443, 2.400)	<0.001
ln.SIRI	2.632 (1.961, 3.533)	<0.001	2.877 (2.086, 3.966)	<0.001	3.084 (2.123, 4.481)	<0.001
Q1 (≤−0.20)	1		1		1	
Q2 (−0.20, 0.21)	2.062 (0.921, 4.617)	0.078	2.171 (0.962, 4.900)	0.062	2.344 (1.008, 5.446)	0.048
Q3 (0.21, 0.76)	3.117 (1.445, 6.727)	0.004	3.288 (1.510, 7.159)	0.003	3.679 (1.635, 8.280)	0.002
Q4 (>0.76)	8.110 (3.907, 16.831)	<0.001	9.302 (4.319, 20.034)	<0.001	9.893 (4.330, 22.600)	<0.001
Linear trend	1.996 (1.609, 2.477)	<0.001	2.074 (1.651, 2.606)	<0.001	2.088 (1.626, 2.681)	<0.001
SII–SIRI pattern						
Low (ln.SII = 5.52, ln.SIRI = −0.39)	1		1		1	
Medium (ln.SII = 6.16, ln.SIRI = 0.40)	3.043 (1.673, 5.534)	<0.001	3.179 (1.740, 5.809)	<0.001	3.693 (1.951, 6.989)	<0.001
High (ln.SII = 7.11, ln.SIRI = 1.43)	8.331 (4.378, 15.856)	<0.001	9.579 (4.822, 19.027)	<0.001	11.261 (5.064, 25.043)	<0.001
Linear trend	1.471 (1.152, 1.879)	0.002	1.463 (1.142, 1.873)	0.003	1.554 (1.185, 2.038)	0.001

The minimally adjusted model adjusted for age, gender, treatment group, and type of operation. All independent variables were adjusted in the fully adjusted model, except for the independent variable of interest itself and the variables required for its calculation.

**Figure 4:**
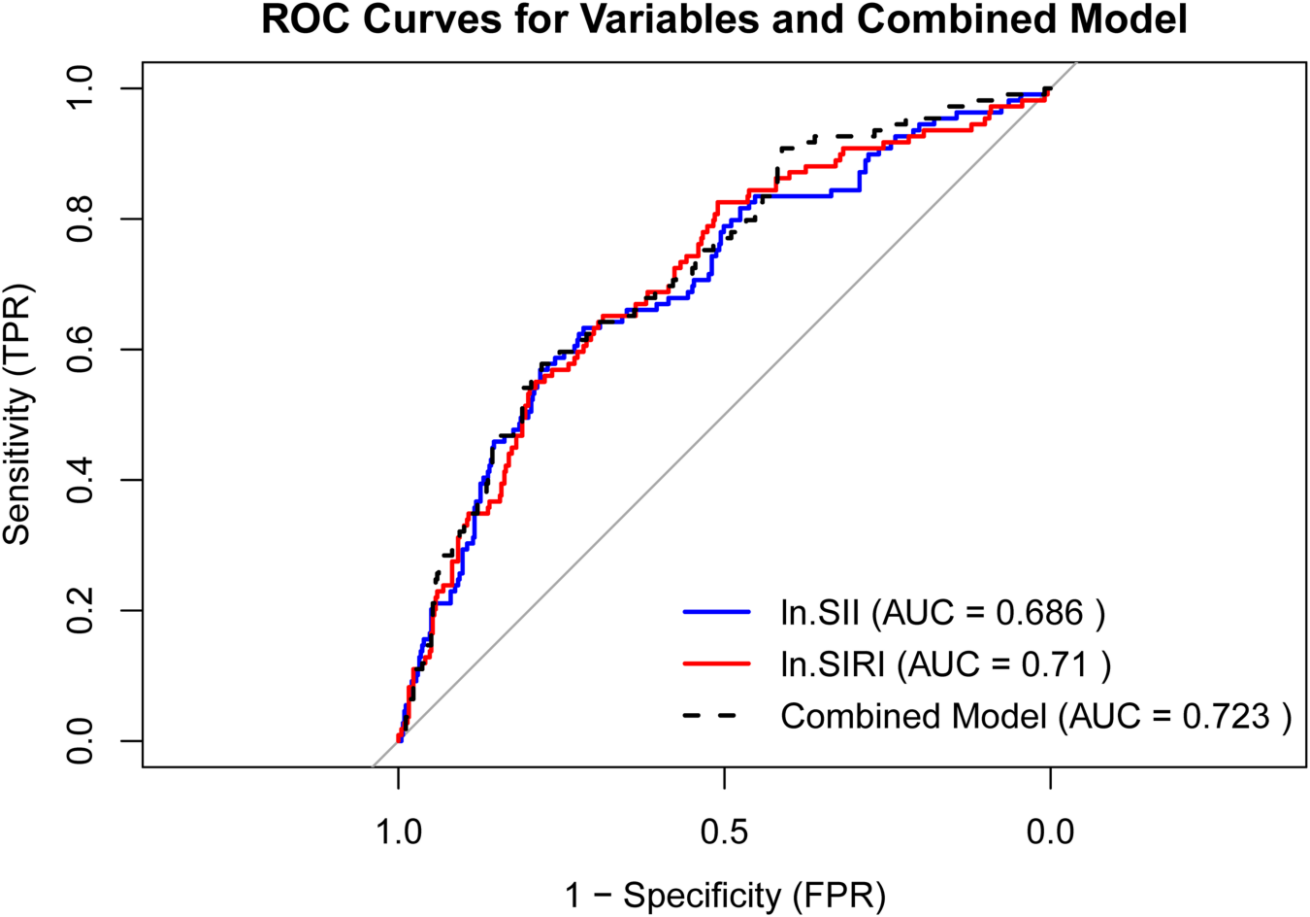
ROC curves for ln.SII, ln.SIRI, and their combined model.

The ROC curves for ln.SII, ln.SIRI, and their combined model are presented in Figure 4, with AUC values of 0.686, 0.71, and 0.723, respectively. The combined model exhibited a diagnostic sensitivity of 83.5% and a specificity of 44.2%. The cutoff values for ln.SII and ln.SIRI were 5.93 (with a sensitivity of 80.4% and a specificity of 46.4%) and 0.10 (with a sensitivity of 81.4% and a specificity of 50.6%), respectively.

### 3.3 Stratified analysis

To further study the roles of potential confounders in the associations of ln.SII/ln.SIRI with frailty progression, we divided the patients into subgroups stratified by gender (male, female), age (≤74, >74 years old), type of operation (joint replacement, lumbar spinal fusion), grip strength decrease (with, without), and treatment group (intervention, observation). We found a consistently positive association between ln.SII/ln.SIRI and frailty progression in all subgroups. However, we did not find any significant interactions between ln.SII/ln.SIRI and those potential confounders (all interaction *p* > 0.05, Table 3).

**Table 3:** Stratified analysis of postoperative frailty progression in older patients after elective orthopedic surgery.

Variables	Stratification	OR (95% CI), *p*	Interaction, *p*
ln.SII			
Gender	Male	2.802 (1.595, 4.922), <0.001	0.493, 0.281
	Female	3.281 (2.044, 5.267), <0.001	
Age	≤74	2.189 (1.237, 3.685), 0.005	1.376, 0.456
	>74	2.882 (1.981, 4.583), <0.001	
Operation	Joint	2.438 (1.568, 3.987), 0.001	1.358, 0.378
	Lumber	3.408 (2.008, 5.581), <0.001	
Grip strength	Normal	2.412 (1.487, 4.296), <0.001	1.511, 0.467
	Decrease	2.976 (1.998, 4.678), <0.001	
Treatment group	Intervention	3.079 (1.923, 4.531), <0.001	1.082, 0.741
	Observation	2.618 (1.671, 4.101), <0.001	
ln.SIRI			
Gender	Male	3.149 (2.470, 4.968), <0.001	0.648, 0.412
	Female	2.231 (1.474, 3.378), <0.001	
Age	≤74	2.621 (1.801, 4.273), 0.001	0.712, 0.453
	>74	2.105 (1.476, 3.147), <0.001	
Operation	Joint	2.315 (1.478, 3.625), 0.001	1.261, 0.498
	Lumber	2.888 (1.906, 4.378), <0.001	
Grip strength	Normal	2.289 (1.378, 3.521), 0.003	1.289, 0.550
	Decrease	2.611 (1.807, 3.808), <0.001	
Treatment group	Intervention	3.011 (2.175, 4.501), <0.001	1.018, 0.864
	Observation	2.963 (1.728, 4.236), <0.001	

## 4 Discussion

Our study innovatively established postoperative frailty progression as a defined clinical endpoint. This outcome not only facilitates evidence-based clinical decision-making but also effectively mitigates potential confounding effects from transient functional fluctuations that may occur during individual follow-up assessments due to factors such as rehabilitation status. Our results revealed that significant changes in the SII–SIRI pattern/SII/SIRI levels are associated with an increased frailty progression risk in older patients (≥65 years) with chronic degenerative orthopedic diseases undergoing elective surgery. We also found that the association between SII/SIRI levels and the occurrence of frailty progression exhibited a non-linear dose–response relationship. ln.SII, ln.SIRI, and their combined model demonstrated considerable predictive value for the postoperative frailty progression. Therefore, our study provides a non-invasive preoperative screening method, monitoring SII and SIRI levels and combining these two indexes in analysis, to identify such patients early and predict postoperative frailty progression.

Frailty has been shown to be strongly correlated with sarcopenia, nutritional status, and age (25, 26). The mechanisms underlying frailty remain unclear, but it is associated with inflammatory responses and immune aging (27, 28). Our study indicates that preoperative frailty is not uncommon among patients undergoing elective orthopedic surgery, with 143 patients (26.2%) identified as frail, surpassing its prevalence in the general older population in our country (29). We suggest that this may be attributed to two main factors: First, patients undergoing orthopedic surgery often experience decreased mobility due to joint and lumbar diseases, increasing their risk of frailty; second, the chronic orthopedic conditions may lead to a state of chronic inflammation or immune activation, facilitating the onset of frailty (15, 30, 31). The relationship between immune system changes and frailty involves multiple pathways. Neutrophils serve as key biomarkers of innate immunity, platelets may contribute to immune function, and monocytes and lymphocytes provide extensive information on adaptive immunity (32, 33). SII and SIRI have demonstrated remarkable efficacy as emerging biomarkers across various diseases (34). These studies underscore the role of managing inflammatory markers in older adults with frailty and suggest that emerging biomarkers such as SII and SIRI may be powerful tools for assessing and managing health in older patients (35, 36). Especially, the SII and SIRI are derived from blood cell counts and are advantageous in that their measurement is low cost, easy, and highly reproducible in laboratory settings (37).

Our study found that, even among patients who underwent elective orthopedic surgery and experienced improvements in mobility, frailty continued to progress in some individuals. According to the fully adjusted model, the patients with the highest levels of SII and SIRI were about 6 times and 9 times more likely to experience frailty progression than patients with the lowest levels of SII and SIRI. Moreover, patients who experienced frailty progression had significantly higher preoperative SII–SIRI/SII/SIRI levels, with a linear relationship between the OR values and these biomarkers. However, in further RCS curve analysis, we discovered that the relationship between ln.SII/ln.SIRI and the frailty progression was more inclined to be non-linear. This is because the probability of frailty progression shows a significant increase among the population with the highest level (Q4) of ln.SII/ln.SIRI (Figure 3, Table 2). Similarly, patients with high SII–SIRI levels were approximately 10 times more likely to experience frailty progression compared with those with low levels. Our findings are consistent with those of a study using data from the NHANES database (20) and may support our hypothesis that patients with high preoperative SII–SIRI levels are more likely to be in a state of chronic inflammation and immune dysfunction. These patients may already be in a latent stage of frailty development before surgery. Although their mobility improved following elective orthopedic surgery, the trauma from surgery further exacerbated their frailty status (38).

The ROC curves and AUC values substantiated the predictive value of ln.SII/ln.SIRI and their combined model for postoperative frailty progression (Figure 4). We also identified cutoff values for ln.SII and ln.SIRI of 5.93 and 0.10, respectively. These cutoff points can assist clinicians in making a quicker and more intuitive assessment of the likelihood of postoperative frailty progression in older patients, who are undergoing elective orthopedic surgery. Although their diagnostic specificity is not high, these findings still highlight the importance of this study, which predicts and assesses the frailty progression in patients through non-invasive perioperative examinations for elective orthopedic surgery. These findings allow clinicians to improve patient outcomes through medical interventions, such as enhancing nutritional status and treating osteoporosis (39).

In the subgroup analysis, we observed a significant positive correlation between ln.SII/ln.SIRI and frailty progression across all subgroups, including gender, age, type of operation, grip strength decrease, and treatment group. Additionally, no interaction effects were found between these variables, which indicates the consistency of our findings across different populations. The oral calcitriol treatment did not have a significant impact on the study outcomes, possibly due to the extended time period required for calcitriol treatment to improve patients’ osteoporotic conditions, which may not be sufficient to significantly affect the frailty status of patients within a 1-year time frame.

This study has certain limitations. First, our study focused on older patients undergoing joint replacement and lumbar spinal fusion, which limits the generalizability of our conclusions to other populations. Second, due to the influence of osteoarthritis on walking activities in our study population, we did not include gait speed, a well-recognized indicator of frailty. In addition, due to the study design, we were not able to include all the factors that have been shown to be associated with frailty. This does not diminish our discovery of the relationship between ln.SII/ln.SIRI and postoperative frailty progression. In future studies, incorporating more relevant variables may enhance the accuracy of the model predicting postoperative frailty progression, and a multicenter design providing external validation may enhance the generalizability of our conclusions.

In conclusion, postoperative frailty progression is not uncommon among older patients undergoing elective orthopedic surgery for chronic degenerative diseases. The SII and SIRI can serve as effective non-invasive preoperative screening tools to identify patients at high risk of frailty progression. This finding provides a feasible approach to help clinicians implement interventions aimed at improving the immune inflammation status of older patients, potentially mitigating frailty progression in this vulnerable population.

## Data Availability

The datasets used and/or analyzed during the current study are available from the corresponding author on reasonable request. Requests to access these datasets should be directed to lxxx-gaoguanghan@163.com.

## References

[1] Dai BZ Zhou LL Mei YJ. Old age security in rural China: there is a long way to go. Chin Med J (Engl). (2013) 126(22):4348–53. 24238527

[2] Davinelli S Corbi G Scapagnini G. Frailty syndrome: a target for functional nutrients? Mech Ageing Dev. (2021) 195:111441. 10.1016/j.mad.2021.111441 33539905

[3] Dent E Martin FC Bergman H Woo J Romero-Ortuno R Walston JD. Management of frailty: opportunities, challenges, and future directions. Lancet. (2019) 394(10206):1376–86. 10.1016/S0140-6736(19)31785-4 31609229

[4] Soto ME Pérez-Torres I Rubio-Ruiz ME Cano-Martínez A Manzano-Pech L Guarner-Lans V. Frailty and the interactions between skeletal muscle, bone, and adipose tissue impact on cardiovascular disease and possible therapeutic measures. Int J Mol Sci. (2023) 24(5):4535. 10.3390/ijms24054534 36901968 PMC10003713

[5] Cappe M Laterre PF Dechamps M. Preoperative frailty screening, assessment and management. Curr Opin Anaesthesiol. (2023) 36(1):83–8. 10.1097/ACO.0000000000001221 36476726 PMC9794163

[6] Mohamed B Ramachandran R Rabai F Price CC Polifka A Hoh D Frailty assessment and prehabilitation before complex spine surgery in patients with degenerative spine disease: a narrative review. J Neurosurg Anesthesiol. (2023) 35(1):19–30. 10.1097/ANA.0000000000000787 34354024 PMC8816967

[7] Kaskirbayeva D West R Jaafari H King N Howdon D Shuweihdi F Progression of frailty as measured by a cumulative deficit index: a systematic review. Ageing Res Rev. (2023) 84:101789. 10.1016/j.arr.2022.101789 36396032

[8] Bhattarai U Bashyal B Shrestha A Koirala B Sharma SK. Frailty and chronic diseases: a bi-directional relationship. Aging Med (Milton). (2024) 7(4):510–5. 10.1002/agm2.12349 39234207 PMC11369349

[9] Urano T Kuroda T Shiraki M. Nutritional and inflammation factors associated with current frailty level and effect of co-morbidities on the progression of frailty. Geriatr Gerontol Int. (2024) 24(6):523–8. 10.1111/ggi.14873 38618879

[10] Soltani A Abolhassani N Marques-Vidal P Aminian K Vollenweider P Paraschiv-Ionescu A. Real-world gait speed estimation, frailty and handgrip strength: a cohort-based study. Sci Rep. (2021) 11(1):18966. 10.1038/s41598-021-98359-0 34556721 PMC8460744

[11] Custodero C Agosti P Anton SD Manini TM Lozupone M Panza F Effect of physical activity intervention on gait speed by frailty condition: a randomized clinical trial. J Am Med Dir Assoc. (2023) 24(4):489–96. 10.1016/j.jamda.2023.01.023 36878264

[12] Kuang K Huisingh-Scheetz M Miller MJ Waite L Kotwal AA. The association of gait speed and self-reported difficulty walking with social isolation: a nationally-representative study. J Am Geriatr Soc. (2023) 71(8):2549–56. 10.1111/jgs.18348 37000466 PMC10524495

[13] Dudzińska-Griszek J Szuster K Szewieczek J. Grip strength as a frailty diagnostic component in geriatric inpatients. Clin Interv Aging. (2017) 12:1151–7. 10.2147/CIA.S140192 28794619 PMC5538538

[14] Ibrahim K Howson FFA Culliford DJ Sayer AA Roberts HC. The feasibility of assessing frailty and sarcopenia in hospitalised older people: a comparison of commonly used tools. BMC Geriatr. (2019) 19(1):42. 10.1186/s12877-019-1053-y 30770722 PMC6377779

[15] Ferrucci L Fabbri E. Inflammageing: chronic inflammation in ageing, cardiovascular disease, and frailty. Nat Rev Cardiol. (2018) 15(9):505–22. 10.1038/s41569-018-0064-2 30065258 PMC6146930

[16] Hu B Yang XR Xu Y Sun YF Sun C Guo W Systemic immune-inﬂammation index predicts prognosis of patients after curative resection for hepatocellular carcinoma. Clin Cancer Res. (2014) 20:6212–22. 10.1158/1078-0432.CCR-14-0442 25271081

[17] Qi Q Zhuang L Shen Y Geng Y Yu S Chen H A novel systemic inﬂammation response index (SIRI) for predicting the survival of patients with pancreatic cancer after chemotherapy. Cancer. (2016) 122:2158–67. 10.1002/cncr.30057 27152949

[18] Dziedzic EA Gąsior JS Tuzimek A Paleczny J Junka A Dąbrowski M Investigation of the associations of novel inflammatory biomarkers-systemic inﬂammatory index (SII) and systemic inﬂammatory response index (SIRI)-with the severity of coronary artery disease and acute coronary syndrome occurrence. Int J Mol Sci. (2022) 23:9553. 10.3390/ijms23179553 36076952 PMC9455822

[19] Xia Y Xia C Wu L Li Z Li H Zhang J. Systemic immune inﬂammation index (SII), system inﬂammation response index (SIRI) and risk of all-cause mortality and cardiovascular mortality: a 20-year follow-up cohort study of 42,875 US adults. J Clin Med. (2023) 12:1128. 10.3390/jcm12031128 36769776 PMC9918056

[20] Zhang H Liu X Wang X Jiang Y. Association of two novel systemic inflammatory biomarkers and frailty based on NHANES 2007–2018. Front Public Health. (2024) 12:1377408. 10.3389/fpubh.2024.1377408 38655524 PMC11036374

[21] Chen LK Woo J Assantachai P Auyeung TW Chou MY Iijima K Asian working group for sarcopenia: 2019 consensus update on sarcopenia diagnosis and treatment. J Am Med Dir Assoc. (2020) 21(3):300–307.e2. 10.1016/j.jamda.2019.12.012 32033882

[22] Ilyas SI Affo S Goyal L Lamarca A Sapisochin G Yang JD Cholangiocarcinoma—novel biological insights and therapeutic strategies. Nat Rev Clin Oncol. (2023) 20(7):470–86. 10.1038/s41571-023-00770-1 37188899 PMC10601496

[23] Ma R Cui L Cai J Yang N Wang Y Chen Q Association between systemic immune inflammation index, systemic inflammation response index and adult psoriasis: evidence from NHANES. Front Immunol. (2024) 13(15):1323174. 10.3389/fimmu.2024.1323174 PMC10896999 38415255

[24] Riley RD Snell KI Ensor J Burke DL Jr HF Moons KG Minimum sample size for developing a multivariable prediction model: PART II - binary and time-to-event outcomes. Stat Med. (2019) 38(7):1276–96. 10.1002/sim.7992 30357870 PMC6519266

[25] Clegg A Young J Iliffe S Rikkert MO Rockwood K. Frailty in elderly people. Lancet. (2013) 381(9868):752–62. 10.1016/S0140-6736(12)62167-9 23395245 PMC4098658

[26] Kirkwood TBL. Understanding the odd science of aging. Cell. (2005) 120(4):437–47. 10.1016/j.cell.2005.01.027 15734677

[27] Weng NP. Aging of the immune system: how much can the adaptive immune system adapt? Immunity. (2006) 24(5):495–9. 10.1016/j.immuni.2006.05.001 16713964 PMC2266981

[28] Duggal NA Upton J Phillips AC Sapey E Lord JM. An age-related numerical and functional deficit in CD19(+) CD24(hi) CD38(hi) B cells is associated with an increase in systemic autoimmunity. Aging Cell. (2013) 12(5):873–81. 10.1111/acel.12114 23755918 PMC3814412

[29] Zeng XZ Meng LB Li YY Jia N Shi J Zhang C Prevalence and factors associated with frailty and pre-frailty in the older adults in China: a national cross-sectional study. Front Public Health. (2023) 11:1110648. 10.3389/fpubh.2023.1110648 37554734 PMC10406229

[30] Tavenier J Margolick JB Leng SX. T-cell immunity against cytomegalovirus in HIV infection and aging: relationships with inflammation, immune activation, and frailty. Med Microbiol Immunol. (2019) 208(3-4):289–94. 10.1007/s00430-019-00591-z 30900090 PMC6635075

[31] Van Sleen Y Shetty SA Van der Heiden M Venema MCA Gutiérrez-Melo N Toonen EJM Frailty is related to serum inflammageing markers: results from the VITAL study. Immun Ageing. (2023) 20:68. 10.1186/s12979-023-00391-3 38012652 PMC10680197

[32] Bonilla FA Oettgen HC. Adaptive immunity. J Allergy Clin Immunol. (2010) 125:S33–40. 10.1016/j.jaci.2009.09.017 20061006

[33] Fest J Ruiter TR Groot Koerkamp B Rizopoulos D Ikram MA van Eijck CHJ The neutrophil-to-lymphocyte ratio is associated with mortality in the general population: the Rotterdam study. Eur J Epidemiol. (2019) 34:463–70. 10.1007/s10654-018-0472-y 30569368 PMC6456469

[34] Fan W Wei C Liu Y Sun Q Tian Y Wang X The prognostic value of hematologic inflammatory markers in patients with acute coronary syndrome undergoing percutaneous coronary intervention. Clin Appl Thromb Hemost. (2022) 28:10760296221146183. 10.1177/10760296221146183 36567485 PMC9806387

[35] Zhao S Dong S Qin Y Wang Y Zhang B Liu A. Inflammation index SIRI is associated with increased all-cause and cardiovascular mortality among patients with hypertension. Front Cardiovas Med. (2022) 9:1066219. 10.3389/fcvm.2022.1066219 PMC9874155 36712259

[36] Zipf G Chiappa M Porter KS Ostchega Y Lewis BG Dostal J. National health and nutrition examination survey: plan and operations, 1999–2010. Vital Health Stat 1. (2013) 56:1–37. 25078429

[37] Xue J Ma D Jiang J Liu Y. Diagnostic and prognostic value of immune/inflammation biomarkers for venous thromboembolism: is it reliable for clinical practice? J Inflamm Res. (2021) 14:5059–77. 10.2147/JIR.S327014 34629886 PMC8494998

[38] Chen L Li X Lv Y Tan X Zhong VW Rong S Physical frailty, adherence to ideal cardiovascular health and risk of cardiovascular disease: a prospective cohort study. Age Ageing. (2023) 52(1):afac311. 10.1093/ageing/afac311 36626327

[39] Hall DE Arya S Schmid KK Carlson MA Lavedan P Bailey TL Association of a frailty screening initiative with postoperative survival at 30, 180, and 365 days. JAMA Surg. (2017) 152(3):233–40. 10.1001/jamasurg.2016.4219 27902826 PMC7180387

